# The Implications of Diagnosis of Small for Gestational Age Fetuses Using European and South Asian Growth Charts: An Outcome-Based Comparative Study

**DOI:** 10.1155/2014/474809

**Published:** 2014-01-27

**Authors:** Gianpaolo Maso, Mathota A. M. M. Jayawardane, Salvatore Alberico, Monica Piccoli, Hemantha M. Senanayake

**Affiliations:** ^1^Department of Obstetrics and Gynecology, Institute for Maternal and Child Health, IRCCS Burlo Garofolo, 34137 Trieste, Italy; ^2^Department of Obstetrics and Gynecology, Faculty of Medicine, University of Colombo, 00800 Colombo, Sri Lanka

## Abstract

The antenatal condition of small for gestational age (SGA) is significantly associated with perinatal morbidity and mortality and it is known that there are significant differences in birth weight and fetal size among different populations. The aim of our study was to assess the impact on outcomes of the diagnosis of SGA according to Bangladeshi and European antenatal growth charts in Sri Lankan population. The estimated fetal weight before delivery was retrospectively reviewed according to Bangladeshi and European growth references. Three groups were identified: Group 1-SGA according to Bangladeshi growth chart; Group 2-SGA according to European growth chart but not having SGA according to Bangladeshi growth chart; Group 3-No SGA according to both charts. There was a difference in prevalence of SGA between Bangladeshi and European growth charts: 12.7% and 51.7%, respectively. There were statistically significant higher rates in emergency cesarean section, fetal distress in labour, and intrauterine death (*P* < 0.001) in Group 1 compared with Group, 2 and 3. No differences of outcomes occurred between Groups 2 and 3. Our study demonstrated that only cases diagnosed as SGA according to population-based growth charts are at risk of adverse outcome. The use of inappropriate prenatal growth charts might lead to misdiagnosis and potential unnecessary interventions.

## 1. Introduction 

It has been recognized that newborns presenting a birth weight below a certain threshold, usually the 10th percentile for the gestational age, have an increased risk of poor outcome as compared to newborns with birth weight appropriate for gestational age (AGA) [1, 2]. Small for gestational age (SGA) is an antenatal diagnosis, which includes fetal growth restriction (FGR) as well as constitutionally small babies. It is well known that both these conditions are significantly associated with perinatal mortality [2–5] and morbidity and fetal distress in labour [6, 7]. Because of its association with adverse outcomes, the prenatal diagnosis of SGA has a significant impact in clinical practice, increasing anxiety among mothers and caregivers and leading to intervention, such as increasing the rates of hospital admission, intensive fetal monitoring, and termination of pregnancy by induction of labour or cesarean delivery, aimed at reducing neonatal complications. Therefore it is understandable how relevant might be an appropriate diagnosis, both to reduce the risk of adverse neonatal outcome and to avoid an overdiagnosis with subsequent unnecessary interventions [7, 8]. In this regard, it is of importance to consider that the differences of ethnicity have a different impact on fetal growth and neonatal birth weight [9–17]. The lack of prenatal ultrasound (US) growth charts in Sri Lanka led local caregivers to use the western references, mainly derived from Caucasian fetuses [18–20]. This approach is continuing, despite the evidence that the birth weight of neonates from populations of the Indian subcontinent is significantly lower than the European newborns [14, 15]. To evaluate the clinical implications of using different prenatal growth charts in Sri Lankan women, we carried out a preliminary pilot study, based on a retrospective observational analysis, to assess the neonatal and pregnancy outcomes of cases having a prenatal diagnosis of SGA according to either Bangladeshi [14] or European growth charts [18–20].

We tested the following hypotheses: (1) the prenatal application of European growth charts rather than Bangladeshi growth references might increase the rate of cases diagnosed as SGA; (2) the outcomes for those cases prenatally identified as SGA according to Bangladeshi references might differ from newborns either classified as AGA according to South Asian and European growth chart or SGA with the European growth reference but not having SGA diagnosis with South Asian growth chart.

## 2. Methods

A retrospective 6-month period analysis of medical records of all deliveries and neonates was carried out in ward 03 Professorial Unit of De Soysa Hospital for Women, Colombo (Sri Lanka). A letter was posted to each of the selected cases to obtain their consent to participate at the study. According to law on privacy, data were anonymized and each patient was assigned a unique identifier. This identifier did not allow tracing the patient's identity and other sensitive data. Ethical clearance was taken from the Ethical Review Committee of the Faculty of Medicine Colombo (ref. number EC/09/045). Fetuses were categorised according to their estimated fetal weight (EFW), based on Hadlock formula [21], on the last US examination before delivery by using Bangladeshi and European growth charts. Patients who refused consent for collection of data, multiple pregnancies, fetuses with diagnosed congenital abnormalities, and cases with uncertain dates and/or time interval between ultrasound evaluation and delivery longer than one week were excluded from the study. Three groups have been identified: Group 1 (G1)-SGA according to the Bangladeshi growth chart; Group 2 (G2)-SGA according to European but not according to Bangladeshi growth charts; Group 3 (G3)-Not having SGA according to any chart.

Diagnosis of SGA was carried out if the EFW for a given gestational age was below the two standard deviations, respectively for Bangladeshi or European growth charts, as defined in the study of Spencer et al. [14]. The outcome of pregnancies of the three groups was herein evaluated considering (1) gestational age at delivery; (2) the mode of delivery; (3) the rate of fetal distress, defined as the presence of either pathological fetal heart rate pattern or presence of meconium stained liquor (grade 3) in labour; (4) perinatal mortality; (5) intrauterine death. Categorical variables was presented as absolute frequencies, percentages and 95% confidence intervals. Difference in means between groups was analyzed using the ANOVA if data were normally distributed, or else with the nonparametric Kruskal-Wallis test. Post hoc analysis was carried out using Bonferroni's correction. Difference in proportion among groups were analyzed using chi-squared test or Fisher exact test as appropriate, and the Bonferroni correction was applied in case of multiple testing. Data was entered and analysed using STATA statistical software package (version 9) and *P* < 0.05 was considered as statistically significant.

## 3. Results

In the study period, information was collected on a total of 1,438 deliveries. 40 multiple pregnancies, 10 newborns with congenital anomalies, and 61 cases with uncertainty of dates were excluded from the analysis, resulting in a study population of 1,327 deliveries. None refused consent for the collection of their data.

The mean age of mothers in G1, G2, and G3 was 28.4, 29.0, and 29.7 years, respectively. There was a statistically significant difference among the three groups (*P* = 0.02). The ethnic composition of the babies was considered according to the parental nationalities. They are comprised of Sinhalese, Tamils, Muslims, and mixed (indicating intermarriages among two of the above nationalities). Distribution of ethnicity in three groups showed no significant differences (*P* = 0.57).

The prevalence of SGA according to the Bangladeshi and European Growth charts is reported in Table 1. The percentages of cases diagnosed as SGA varied according to different references: 12.7% (95% confidence interval, 10.9–14.5) and 51.7% (95% confidence interval, 47.9–55.4) according to the Bangladeshi (G1) and European Growth charts (G1 plus G2), respectively. The percentage of SGA diagnosed in the European growth chart, not having SGA according to Bangladeshi growth reference (G2), was 39.0% (95% Confidence Interval, 36.4–41.6). The rate of newborns identified as AGA by both charts was 48.3% (95% Confidence Interval, 45.6–50.9).

The rate of preterm delivery (gestational age less than 37 weeks) varied among the three groups as follows: 30.4% in G1, 7.3% in G2, and 12.2% in G3.

There was a statistically significant difference in the distribution of period of gestation at delivery among the three groups (*P* < 0.001). There were no differences between G2 and G3 (*P* = 0.06) (Table 1).

In regard to the mode of delivery, chi-square test showed overall different rates among the three groups (*P* < 0.001). However this difference was not statistically significant between G2 and G3 (*P* = 0.59). Specifically, cases diagnosed as SGA according to both charts (G1) had lower and higher rates of spontaneous vaginal and emergency cesarean delivery rates, respectively (Table 1).

When comparing the proportions of vaginal births in G1 with G2 and G3, there was a statistically significant lower rate of spontaneous vaginal birth in G1 (G1 versus G2, *P* = 0.002; G1 versus G3, *P* = 0.003). There were no differences between G2 and G3 (G2 versus G3, *P* = 0.28).

As for the proportion of elective cesarean section rates, there were no statistically significant differences between G1 and G2 or G3 (G1 versus G2, *P* = 0.39; G1 versus G3, *P* = 0.47). No differences were found between G2 and G3 (G2 versus G3, *P* = 0.37).

The rates of emergency cesarean section rates in labour were significantly higher in G1 than in G2 and G3 (G1 versus G2, *P* < 0.001; G1 versus G3, *P* < 0.001). However there was no statistically significant difference in the proportions of emergency cesarean section rates in G2 and G3 (G2 versus G3, *P* = 0.18).

In regard to the prevalence of fetal distress during labour, there was a significant difference among the three groups (*P* < 0.001). However this difference was not statistically significant between G2 and G3 (*P* = 0.79), leading to the conclusion that the difference was due to the higher rate of fetal distress during labour in G1 (Table 1).

The percentage of occurrence of intrauterine deaths was 3.6% (6 cases), 0.2% (1 case), and 0.5% (3 cases) in G1, G2 and G3, respectively. Although there was an apparent increase in intrauterine death rates in G1, the low prevalence of cases did not allow applying statistical tests to calculate the significance. Therefore G2 and G3 were added together as shown in Table 2.

After this analysis, there was a significant difference between G1 compared with G2 and G3 (*P* < 0.001). Therefore there was a significant higher rate of intrauterine death among babies having SGA according to Bangladeshi growth chart.

There were a total of 8 perinatal deaths during the study period of 6 months in ward 3, 2 perinatal deaths in Group 1, 3 in Group 2, and 3 in Group 3, accounting for 1.2%, 0.6%, and 0.5%, respectively. However statistical tests could not be applied since the numbers were small, to identify any significant difference.

## 4. Discussion

The diagnosis of SGA is made using growth charts during antenatal period and it is commonly based on the estimated fetal weight less than a defined percentile for a determined gestational age. Many causes might affect the potential of fetal growth (i.e., placenta insufficiency, somatic malformations, genetic disorders, and maternal metabolic diseases) and it is widely acknowledged that parental characteristics and ethnicity have a primary influence in determining the “normal” fetal growth and neonatal birth weight [2]. The clinical implications of a correct prenatal diagnosis of SGA have a significant impact in clinical practice. First, it allows to identify the fetuses that are at risk for adverse outcomes, needing close surveillance; second it reduces unnecessary interventions and worrying examinations due to wrongfully assumed fetal growth restriction [2, 22–24].

Due to the lack of availability of growth charts derived from local population, European growth charts are commonly used in Sri lankan setting for the diagnosis of SGA.

Our pilot study demonstrates that the assessment of fetal growth in Sri lankan population using western growth references is clinically misleading.

Our study indicated that applying Bangladeshi growth references to Sri lankan population, the rate of pregnancies affected by SGA is reduced from 51.75% to 12.7%, as reported in other communities. These data are in agreement with the results of other studies. Spencer et al. found that the abdominal circumference and estimated fetal weight in Bangladeshi population are significantly smaller than in Anglo-Saxon population, even if they maintained similar growth patterns in the third trimester [14]. Meire and Farrant observed that the mean birth weight of Indian newborns was 340 grams less than that of the European controls [15]. Kinare et al. observed that fetal size was smaller in a rural Indian population than in European or urban Indian populations with significant differences in fetal biometric measurements. [16]. As observed by Spencer et al., the smallness of Indian fetuses appeared to be related mainly to a smaller abdominal circumference, showing a normal growth pattern significantly below the median for the European growth charts. All these studies focused their attention on differences between western and South Asian fetal growth references. However they did not assess the outcome of pregnancy according to the different fetal growth curves. In this regard, our study is the first that determined the clinical significance of using different ethnic-based fetal growth references. Our findings are of epidemiological interest and, most importantly, are clinically meaningful. Only cases diagnosed as SGA by using South Asian references showed higher incidence of emergency cesarean section, distress in labour, and intrauterine death, demonstrating that, in Sri lankan population, the Bangladeshi growth chart is a better predictor of poor outcome if compared to European references. Moreover our data showed that applying European fetal growth charts to Sri lankan population led to an incorrect assessment of fetal growth with an over diagnosis of SGA in 39% of the cases. The clinical management of SGA according to either Bangladeshi or European references was beyond the scope our study. However, considering the gestational age at delivery and the rate of elective cesarean section, it might be postulated that the local caregivers are aware of the limitations of European growth charts in the assessment of fetal growth. In this context it might be postulated that they do not intervene on the basis of a diagnosis of SGA made only with these charts. Despite these strengths, we are aware that our study has some limitations. First, it was carried out retrospectively, not taking into account other variables of neonatal outcome. However the aim of our study was to obtain evidences to plan a multicentre study to prospectively collect data on neonatal mortality and morbidity based on fetal growth charts for the Sri lankan population. Second, the study was done in a single unit in tertiary care centre in Colombo, where there is a high prevalence of high risk pregnancies. Therefore it may not be representative of national population. Third, we did not differentiate the condition of SGA from fetal growth restriction. However we focused our attention only on SGA diagnosis, because of the ambiguity of current definitions of fetal growth restriction [24].

## 5. Conclusion

In conclusion we are aware that the best option to evaluate fetal growth is based on the customized approach [25]. However, in the clinical setting where a lack of resources or organizational reasons do not allow this approach, specific population-based antenatal references are recommended to properly assess the fetal growth. In this regard, our study demonstrated that a prospective multicentre evaluation to develop specific fetal ultrasonographic biometric references for Sri lankan population might be justified, taking into account the relevant concepts of fetal growth and the recommended methodology of creating prenatal size charts [2, 25, 26]. In this context, the clinical usefulness of curves related to specific population and derived from prognosis-based references will allow estimating the risk of adverse outcomes, reducing the need for inappropriate interventions [24].

## Figures and Tables

**Table 1:** Prevalence, gestational age at delivery, mode of delivery, and occurrence fetal distress in labour among the study groups (Group-1: SGA according only to Bangladeshi growth chart; Group-2: SGA according to European grow chart and AGA according to Bangladeshi growth chart; Group-3: AGA according to both growth chart).

	Group-1		Group-2		Group-3	
	*n*	%	*n*	%	*n*	%
Prevalence	168	12.7	518	39.0	641	46.3
Gestational age at delivery^∗§^						
<37	51	30.4	38	7.3	78	12.2
≥37 weeks	117	69.6	480	92.7	563	87.8
Mode of delivery**						
SVD	87	53.0	354	68.9	424	66.6
ELCS	26	15.9	78	15.2	92	14.4
EMCS	45	27.4	59	11.5	89	14.0
AVD	6	3.6	23	4.4	32	5.1
Fetal distress^†‡^						
Yes	31	18.5	36	6.9	42	6.6
No	137	81.5	482	93.1	599	93.4

Footnotes: SGA: small for gestational age, AGA: appropriate for gestational age, SVD: spontaneous vaginal delivery; ELCS: elective cesarean section; EMCS: emergency cesarean section; AVD: assisted vaginal delivery.

*P < 0.001 for all comparison; ^§^ *P* = 0.06 G2 versus G3; **see text for statistical differences (12 cases had not indicated their mode of delivery); ^†^ *P* < 0.001 for all comparisons; ^‡^ *P* = 0.79 G2 versus G3.

**Table 2:** Distribution of occurrence of intrauterine death between Group 1 and Groups 2-3 (Group-1: SGA according only to Bangladeshi growth chart; Group-2: SGA according to European grow chart and AGA according to Bangladeshi growth chart; Group-3: AGA according to both growth chart).

Intrauterine death*	Group-1		Group-2/3	
	*n*	%	*n*	%
Yes	6	3.6	4	0.3
No	162	96.4	1155	99.7

Total	168	100.0	1159	100.0

*P < 0.001.
